# Penis Amputation

**Published:** 1896-04

**Authors:** Ramon Guiteras

**Affiliations:** New York


### PENIS AMPUTATION.

By RAMON GUITERAS, M. D., New York.

I shall consider simply the anterior operations and not the so-called extirpations. Many methods of performing this operation have been devised, showing that the results are not satisfactory. Among the various operations are amputations by ligature ecraseur, galvano-cautery and galvano-ecraseur, and by the knife. The various writers agree that the urethra should be cut longer than the corpora cavernosa. The principal complications of the operation are hemorrhage, retraction of the orifice of the divided urethra within the stump, contraction of the urethral orifice by cicatricial tissue, and the wetting of the wound by the urine. To overcome the hemorrhage it is necessary to tie a rubber band or catheter about the base of the organ until the amputation has been completed, when the dorsal arteries and those of the cavernosa are to be ligated. The second complication can be guarded against by cutting the urethra half an inch longer than the corpora cavernosa. Surgeons generally seek to avoid cicatricial contraction by slitting up the urethra on its dorsal aspect and stitching the mucous membrane to the skin. This certainly makes a larger orifice, but the urethra is more or less distorted and a still longer stricture is liable to occur there. I do not think that any device can be resorted to by which the orifice will not contract, and that if this method be followed there will be stricture of the orifice extending upward for half an inch. The simple union of the skin seems to me to be better; then if stricture occurs the meatus can be divided. Wetting of the parts with urine always interferes with union. It has been advised to leave a soft catheter in the bladder for two days, but it is an inconvenient method. If it is employed the catheter should be plugged and the bladder only evacuated about every four hours. The retention of the catheter by its pressure tends to cause a slough about the sutures. It is advisable to keep the parts clean by frequent washing with boric acid.

The following is the technique I recommend: The parts having been thoroughly cleansed surgically, a rubber band is tied about the base of the organ and the circular incision is made through the integument and the latter dissected back three-quarters of an inch. A No. 20 French sound is passed into the urethra and held so that the penis is at right angles to the body. A straight bistoury is then inserted with the cutting edge pointing upward just above the point where the flap is rolled back. It is worked behind the urethra between the corpora cavernosa until it comes out at the corresponding point on the other side. The knife is then turned and the corpora cavernosa are divided. They are then dissected away from the urethra for half an inch, when the knife is again turned and brought out through the urethra. The operation really consists in an amputation of the anterior part of the organ, leaving a stump with the urethra half an inch longer and the integument three-quarters of an inch longer than the remainder. The dorsal arteries and the arteries of the corpora cavernosa and the artery of the septum are ligated and oozing controlled by the application of hot water and the pressure of the skin flaps and bandages. Fine silk sutures are passed through the integument and urethra at each extremity of the canal. These are then tied. The integument above and below the urethra is then sewn together with a continuous silk suture. The urethra is next put upon the stretcher and four sutures of fine silk are passed through the integument and urethra. The sutures are pulled up in the middle, cut and tied on either side. Thus the skin and urethra are held together by eight sutures. The parts are then washed with sterilized water and a sound passed through the new canal into the bladder. A No. 15 French catheter is then introduced and left for a few days. The catheter is plugged and this is removed every four hours to allow the urine to escape. The parts are supported by a T bandage. Extirpation of the inguinal glands is performed in the case of amputation for malignant disease.—*Medical Sentinel.*

